# A Novel Mouse Model of *MYO7A* USH1B Reveals Auditory and Visual System Haploinsufficiencies

**DOI:** 10.3389/fnins.2019.01255

**Published:** 2019-11-22

**Authors:** Kaitlyn R. Calabro, Sanford L. Boye, Shreyasi Choudhury, Diego Fajardo, James J. Peterson, Wei Li, Sean M. Crosson, Mi-Jung Kim, Dalian Ding, Richard Salvi, Shinichi Someya, Shannon E. Boye

**Affiliations:** ^1^Department of Ophthalmology, University of Florida, Gainesville, FL, United States; ^2^Department of Pediatrics, University of Florida, Gainesville, FL, United States; ^3^Department of Aging and Geriatric Research, University of Florida, Gainesville, FL, United States; ^4^Department of Communicative Disorders and Sciences, The State University of New York at Buffalo, Buffalo NY, United States

**Keywords:** retina, cochlea, Usher syndrome, myosin-7A, vision, hearing

## Abstract

Usher’s syndrome is the most common combined blindness–deafness disorder with USH1B, caused by mutations in *MYO7A*, resulting in the most severe phenotype. The existence of numerous, naturally occurring *shaker1* mice harboring variable *MYO7A* mutations on different genetic backgrounds has complicated the characterization of MYO7A knockout (KO) and heterozygote mice. We generated a novel MYO7A KO mouse (*Myo7a*^–^*^/^*^–^) that is easily genotyped, maintained, and confirmed to be null for MYO7A in both the eye and inner ear. Like USH1B patients, *Myo7a*^–^*^/^*^–^ mice are profoundly deaf, and display near complete loss of inner and outer cochlear hair cells (HCs). No gross structural changes were observed in vestibular HCs. *Myo7a*^–^*^/^*^–^ mice exhibited modest declines in retinal function but, unlike patients, no loss of retinal structure. We attribute the latter to differential expression of MYO7A in mouse vs. primate retina. Interestingly, heterozygous *Myo7a*^+^*^/^*^–^ mice had reduced numbers of cochlear HCs and concomitant reductions in auditory function relative to *Myo7a*^+/+^ controls. Notably, this is the first report that loss of a single *Myo7a* allele significantly alters auditory structure and function and suggests that audiological characterization of USH1B carriers is warranted. Maintenance of vestibular HCs in *Myo7a*^–^*^/^*^–^ mice suggests that gene replacement could be used to correct the vestibular dysfunction in USH1B patients. While *Myo7a*^–^*^/^*^–^ mice do not exhibit sufficiently robust retinal phenotypes to be used as a therapeutic outcome measure, they can be used to assess expression of vectored *MYO7A* on a null background and generate valuable pre-clinical data toward the treatment of USH1B.

## Introduction

Usher syndrome is the most frequent cause of combined deafness–blindness (Smith et al., 1994). Recessive mutations in *MyosinVIIa* (*MYO7A*) are associated with the most common form, USH1B (Weil et al., 1995; Astuto et al., 2000; Bharadwaj et al., 2000; Ouyang et al., 2005). Patients are born profoundly deaf, have vestibular dysfunction, and exhibit progressive loss of vision in their first decade (Petit, 2001). *MYO7A* encodes an unconventional myosin expressed in sensory hair cells (HCs) of the inner ear and the retina. It is expressed early in auditory and vestibular HC development and is required for proper differentiation and development of stereocilia bundles (Roccio et al., 2018). It is therefore unsurprising that mutations rendering MYO7A dysfunctional or absent lead to congenital deafness. The absence/malformation of cochlear HCs suggests that gene supplementation will not be an effective strategy for restoring hearing to USH1B patients. Fortunately, their hearing loss can be addressed with cochlear implantation. In contrast to the structural malformations observed in the inner ear from birth, clinical studies show that patients display progressive retinal degeneration, but can retain both central and peripheral islands of structurally and functionally normal retina into the third decade of life (Jacobson et al., 2008, 2009, 2011; Sumaroka et al., 2016). It is therefore possible that gene supplementation targeted to these preserved regions will be an effective strategy for restoring/preserving vision in these patients.

Development of a retinal gene therapy for USH1B has been hampered by the lack of animal models that faithfully replicate human retinal disease. Although currently available mouse models mimic the hearing loss and vestibular dysfunction seen in patients, they do not exhibit progressive retinal degeneration/dysfunction (el-Amraoui et al., 1996; Self et al., 1998; Libby and Steel, 2001; Lillo et al., 2003). Only subtle retinal phenotypes have been reported. These include (1) modest reductions in retinal function (if backcrossed onto an albino background), defective melanosome transport in the apical RPE, and impaired rhodopsin (RHO) transport through photoreceptor connecting cilia (Williams, 2008; Colella et al., 2013; Trouillet et al., 2018). We, and others, have corrected these subtle phenotypes following treatment with either fragmented or dual AAV-MYO7A vectors (Liu et al., 1997, 1998, 1999; Colella et al., 2013; Lopes et al., 2013). Differences in retinal disease presentation across species are likely related to differential expression of MYO7A in mouse vs. primate retina. In macaques and humans, MYO7A is predominantly localized to photoreceptor inner segments (el-Amraoui et al., 1996; Sahly et al., 2012). In contrast, it is expressed predominantly in mouse retinal pigment epithelium (RPE) (el-Amraoui et al., 1996; Sahly et al., 2012). One explanation for this difference, albeit one that is still debated, relates to structural differences between mouse and primate photoreceptors (Sahly et al., 2012; Volland et al., 2015). Analysis of the apical region of macaque and human photoreceptor inner segments revealed the presence of calyceal processes, within which MYO7A was localized (Sahly et al., 2012). These structures were absent in mouse retina (Sahly et al., 2012), an observation that may explain why mouse models fail to recapitulate the robust retinal degeneration/dysfunction seen in USH1B patients.

The purpose of our study was to generate and characterize a novel, traditional MYO7A knockout (KO) mouse (*Myo7a^–/–^*) that could be more easily genotyped, maintained, and studied relative to existing strains (Gibson et al., 1995; Libby and Steel, 2001; Peng et al., 2011). We completed a full characterization of retinal and cochlear HC structure/function, and vestibular HC structure in *Myo7a^–/–^*, *Myo7a^+/–^*, and *Myo7a*^+/+^ littermates. We further investigated the role of MYO7A in RHO trafficking and the differential expression of MYO7A in mouse vs. primate retinas. Results of this study further inform the USH field regarding the impact of complete vs. partial loss of MYO7A on multiple sensory systems, and will aid in the development of therapies aimed to preserve the function of these systems in USH1B patients.

## Materials and Methods

### Animal Ethics Statement

All mice were bred and maintained at the University of Florida Health Science Center Animal Care Services Facility under a 12 h/12 h light/dark cycle. Food and water were available *ad libitum*. All experiments were approved by the University of Florida’s Institutional Animal Care and Use Committee and were conducted in accordance with the ARVO Statement for the Use of Animals in Ophthalmic and Vision Research and within National Institute of Health regulations. Mice were anesthetized for all in-life experiments with ketamine (100 mg/kg) and xylazine (10 mg/kg) by intraperitoneal injection.

### Generation of *Myo7a^–/–^* Mice

**FIGURE 1:**
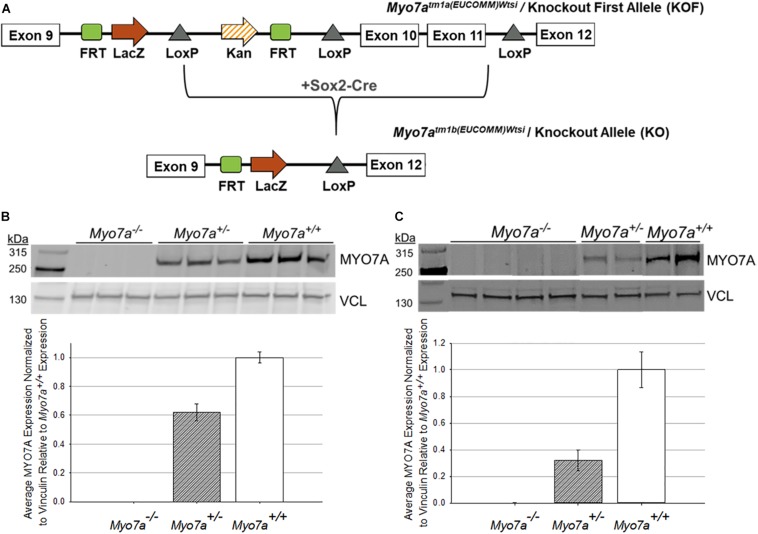
Generation and confirmation of MYO7A knockout in *Myo7a*^–/–^ mice. **(A)** Schematic of how *Myo7a*^–/–^ mice were generated using Cre-lox recombination. Exons 10 and 11 of *Myo7a* were deleted by breeding *Myo7a^TM1*a(EUCOMM)Wtsi*^* mice with a Sox2-Cre deleter line, creating the *Myo7a^TM1*b(EUCOMM)Wtsi*^* or knockout allele. Immunoblotting was performed to compare differences in MYO7A expression in 4-month-old *Myo7a*^+/+^, *Myo7a*^+/–^, and *Myo7a*^–/–^ **(B)** eyes (*n* = 3 per genotype) and **(C)** cochlea (*n* = 2–4 per genotype). Protein levels were quantified and compared using *Fiji*. MYO7A expression was normalized to vinculin and analyzed relative to MYO7A expression in *Myo7a*^+/+^ mice. FRT, flippase recognition target; Kan, kanamycin; kDa, kilodaltons; VCL, vinculin.

Mouse ES cells, created as part of the International Knockout Mouse Consortium (IKMC), were acquired from the Sanger Institute, and mice were generated at Jackson Laboratories (Ryder et al., 2013). The targeted trap “tm1a” cassette was inserted between exons 9 and 10 of the *Myo7a* gene (Skarnes et al., 2011). This cassette contains a flippase recognition target (FRT) followed by lacZ sequence and a loxP site (Figure 1A). The loxP site is followed by kanamycin/neomycin sequence under the control of the human beta-actin promoter, an SV40 polyA, a second FRT site, and a second loxP site. A third loxP site was inserted between Exons 11 and 12 of *Myo7a*. The critical exons were thus flanked by loxP sites. ES cells were implanted in C57BL/6N mice to create the Myo7atm1a(EUCOMM)*^*Wtsi*^* mouse. C57BL/6N mice carry the retinal disease-causing rd8/Crb1 genes (Mattapallil et al., 2012). The *Myo7a*^TM^*^1*a(EUCOMM)Wtsi*^* mice were backcrossed on C57BL/6J mice and screened to ensure removal of the rd8/Crb1 alleles. Resultant weanlings were then crossed with a Sox2-Cre deleter line to create *Myo7a*^TM^*^1*b(EUCOMM)/Wtsi*^*, which will from here on be referred to as *Myo7a^–/–^* mice. Colony founders were screened and confirmed to have the *RPE65-*L450M mutation as previously described (Wu et al., 2009). Founders were then backcrossed on a C57BL/6J background for at least five generations prior to use in experiments. The *Myo7a^–/–^* colony was maintained by breeding heterozygous females with homozygous males. *Myo7a*^+/+^ (wild-type) controls were generated by breeding heterozygous females with heterozygous males. All mice were genotyped prior to use with the following two primer sets: (1) *Myo7a* WT Allele: *Myo7a* Forward (GGG AGA GAA AC AGG GTG TG) + *Myo7a* WT Reverse (AAG CTG GAC TCT CTG GTG GC) and (2) *Myo7a* KO Allele: *Myo7a* Forward + *Myo7a* KO Reverse (TCG TGG TAT CGT TA GCG CC). *Myo7a* WT primers produce an amplicon of 360 bp, and the *Myo7a* KO primers produce an amplicon of 178 bp. *Myo7a* WT was amplified using the following conditions: (1) initial denaturation at 95°C for 2 min; (2) 34 cycles of 95°C denaturation for 30 s, 66°C annealing for 30 s, and 72°C elongation for 45 s; and (3) final elongation at 72°C for 5 min. The *Myo7a* KO primer set was run using identical PCR settings, with the exception of a 63°C annealing temperature. *LacZ* also served as a genotyping target for the KO allele using the following primers: *LacZ* Forward (ACA TCG GGC AAA TAA TAT CG) + *LacZ* Reverse (ATC ACG ACG CGC TGT ATC). *LacZ* sequence was amplified using the PCR settings described above with an annealing temperature of 56°C.

### Generation of *Myo7a:RHOGFP* Mice

Male *Myo7a^–/–^* mice were crossed with females homozygous for the *hrhoG* allele, a knock-in human rhodopsin–GFP fusion (*RHOGFP*) that were maintained on an albino C57BL/6J background (Chan et al., 2004). All mice were genotyped prior to use for both the *Myo7a* and *RHOGFP* genes. *RHOGFP* alleles were amplified as previously described, producing a 194 bp amplicon for the WT RHO allele or a 922 bp amplicon for the *RHOGFP* allele (Chan et al., 2004). *RHOGFP* mice were also crossed to transgenic P23H mice containing the entire human RHO gene to serve as controls (Mao et al., 2011). These mice will hereon be referred to as P23H^+^*:RHOGFP^+/–^*. *P23H* was amplified using the following primers: *P23H* Forward (ACC TCT TTC CAC TAC AGG CC) + *P23H* Reverse (CCT GGT GAT TAT GCC CAA CG). PCR settings were the same as those described above, with an annealing temperature of 60°C. *P23H* primers produce a 290 bp amplicon when the *P23H* allele is present.

### Western Blots

Eyes from *Myo7a*^+/+^, *Myo7a^+/–^*, and *Myo7a^–/–^* were enucleated, and cochleas were extracted from the temporal bones. Both male and female mice were used for western blot analysis (*n* = 3–4/genotype). Whole eyes and cochleas were homogenized in RIPA lysis buffer (Santa Cruz, sc-364162). Protein concentration within each sample was determined using BCA assay (Thermo Scientific, 23225). Equal amounts of protein from each sample were loaded on 8–12% Tris-acetate gels (Invitrogen, EA0375) and run for approximately 1 h at 120 V (Invitrogen, A25977). Gels were transferred to a PVDF membrane using a semi-dry system (BioRad, 1704150). The membrane was blocked for 1 h at room temperature in Licor blocking buffer (927-40000) and incubated at 4°C overnight with primary antibodies [MYO7A: Santa Cruz, sc74516, 1:500; Vinculin (VCL): Santa Cruz, sc73614, 1:1000]. Secondary antibody (Licor, 926-68072) incubation took place the following day at room temperature for 1 h. Membranes were then visualized on a Licor Odyssey CLx Imager. Protein quantification was performed by measuring the mean gray for each sample using the open source *Fiji* software (Schindelin et al., 2012). Protein quantification was normalized to VCL in each sample and written as values relative to MYO7A expression in C57BL/6J mice.

Neural retina and RPE were manually separated from the eyes of 5-month-old male C57BL/6J mice (*n* = 4) and a 5-year-old male macaque (*Macaca fascicularis*). Tissues were collected and flash-frozen. MYO7A expression in mouse and non-human primate (NHP) tissues was determined with Western Blot, as described above. The membrane was incubated at 4°C overnight with primary antibodies (MYO7A: Santa Cruz, sc74516, 1:500; β-actin: Santa Cruz, sc47778, 1:1000).

### Auditory Brainstem Response Testing

Auditory brainstem response (ABR) thresholds were measured in 5-month-old mice with a tone burst stimulus at 8, 16, and 32 kHz using an ABR recording system (Tucker-Davis Technologies) as previously described (Han et al., 2016; White et al., 2017). Mice were anesthetized and subdermal needle electrodes were placed at the vertex (active), ipsilateral ear (reference), and contralateral ear (ground). At each frequency, the sound level was reduced in 5–10 dB increments from 90 to 10 dB sound pressure level (SPL). A hearing threshold was defined as the lowest level that produced a noticeable ABR as previously described (Han et al., 2016; White et al., 2017). Both male and female mice were used for ABR threshold assessments (*n* = 5–6/genotype). Two-way analysis of variance (ANOVA) with Bonferroni’s multiple comparisons tests (GraphPad Prism 7) were used to analyze ABR thresholds in *Myo7a*^+/+^, *Myo7a^+/–^*, and *Myo7a^–/–^* mice.

### Structural Assessment of Cochlear Hair Cells

Following ABR testing, mice were sacrificed by cervical dislocation. Temporal bones were excised from the head and divided into cochlear and vestibular parts as previously described (Han et al., 2016; White et al., 2017). The excised cochlea was immersed in 10% formalin for 1 day and shipped to the University at Buffalo for analysis. The fixed cochlea was stained with hematoxylin and the cochlear surface preparation was performed as previously described (Ding et al., 2016). HCs were observed in the middle turn of cochlear basilar membrane using a 200× objective as previously described (Ding et al., 2016). For cochleograms, the numbers of inner hair cells (IHCs), first-row outer hair cells (OHC1), second-row outer hair cells (OHC2), and third-row outer hair cells (OHC3) were counted over 0.24 mm intervals from the apex to the base of the cochlea under the microscope at 40× magnification as previously described (Ding et al., 2016). The counting results were then calculated to compute a cochleogram and frequency-place map for mouse cochlea that shows the percentage of missing IHC and OHC as a function of percentage distance from the apex of the cochlea as previously described (Muller et al., 2005; Ding et al., 2016).

### Structural Assessment of Spiral Ganglion Neurons and Stria Vasicularis

Excised cochlea was immersed in 4% paraformaldehyde in PBS for 1 day, decalcified in 10% EDTA, pH 7.4, in PBS for 5–7 days, and embedded in paraffin. The paraffin-embedded cochlear specimens were sliced into 5 μm sections, mounted on silane-coated slides, stained with hematoxylin and eosin (H&E), and observed under a light microscope (Leica Microsystems). Spiral ganglion neurons (SGNs) and stria vascularis (SV) were observed in the apical, middle, and basal cochlear tissues using a 40× objective as previously described (Han et al., 2016; White et al., 2017).

### Structural Assessment of Vestibular Hair Cells

The excised vestibular apparatus was immersed in 10% formalin for several days. The vestibular end organs were placed in PBS and carefully dissected out as previously described (Zhang et al., 2003). The superior, horizontal, and posterior portions of the vestibular apparatus were opened. The saccule and utricle were separated from surrounding tissue and the overlying otoconia on the saccule and utricle were removed to expose the macula of the saccule and utricle. The ampulla in each of the semicircular canals was separated from the surrounding connective tissue. The maculae and ampullae were stained with hematoxylin and mounted as surface preparation in glycerin on glass slides. Surface preparations were viewed with an Axioskop light microscope (Carl Zeiss) and photographed with a digital camera (SPOT Insight, Diagnostic Instruments Inc.) and processed with software (PowerPoint 2010).

### Optical Coherence Tomography

Prior to Optical Coherence Tomography (OCT) analysis, pupils of mice were dilated (atropine 1%, phenylephrine 2.5%). Mice were anesthetized, and retinal cross-section images were obtain with a spectral domain (SD) OCT system (Bioptigen, Inc., Durham, NC, United States). Post-imaging ONL measurements were performed with commercial software (InVivoVue, Bioptigen, Inc.), as previously described (Pang et al., 2011). Equal numbers of males and females were used for each genotype (*n* = 10–11/genotype). Two-way ANOVA with Bonferroni’s multiple comparisons tests were used to analyze OCT measurements in *Myo7a*^+/+^, *Myo7a^+/–^*, and *Myo7a^–/–^* mice. Significance was defined as a *P*-value <0.05.

### Electroretinogram

Following overnight dark adaptation, mice were dilated (phenylephrine HCL-tropicamide 1%) and anesthetized. Full-field electroretinograms (ERGs) were recorded using a Celeris unit (Diagnosys LLC) according to methods previously described, with minor modifications (McCullough et al., 2019). Briefly, scotopic ERGs were elicited at intensities ranging from 0.025 to 2.5 cds/m^2^, with interstimulus intervals of 30 s and averaged from five measurements at each intensity. Mice were then light-adapted to a 30 cds/m^2^ white background light for 5 min. Photopic responses were elicited, with intensities ranging from 1.25 to 25 cds/m^2^. Fifty responses with interstimulus intervals of 0.4 s were recorded in the presence of a 20 cds/m^2^ white background and averaged at each intensity. Equal numbers of males and females were used for each genotype (*n* = 10–11/genotype). Two-way ANOVA with Bonferroni’s multiple comparisons were used to analyze ERG amplitudes in *Myo7a*^+/+^, *Myo7a^+/–^*, and *Myo7a^–/–^* mice. Significance was defined as a *P*-value <0.05.

### Retinal Tissue Processing and Microscopy

*Myo7a^–/–^*:*RHO-GFP^+/–^* mice were sacrificed between 1 and 6 months of age. Eyes were enucleated and fixed in formalin overnight, then dehydrated using a Tissue-Tek VIP6 automatic processor. Eyes were then embedded in paraffin using a Histocentre embedding station. Paraffin-embedded eyes were sectioned (7 μm) using a Leica microtome (Reichert-Jung BioCut 2030). Paraffin sections were then re-hydrated, and counter-stained with 4′,6-diamidino-2-phenylindole (DAPI) (1:10,000) for 5 min at room temperature. Both DAPI and native GFP expression from RHOGFP were visualized using a Leica TCS SP8 confocal microscope. *Myo7a*^+/+^:*RHO-GFP^+/–^*, *Myo7a^+/–^*:*RHO-GFP^+/–^*, and *P23H^+/–^*:*RHO-GFP^+/–^* mice served as controls. Equal numbers of males and females were used for each genotype (*n* = 4 mice/genotype per time point). Exposure and gain settings remained constant for all images.

## Results

### Design and Confirmation of *Myo7a^–/–^* Mice

Traditional *Myo7a* KO mice (*Myo7a^–/–^*) were generated using previously described methods (Ryder et al., 2013). Critical exons 10–11, which correspond to the motor and actin-binding domain of MYO7A, were deleted using Cre-lox recombination (Figure 1A). This large deletion allows for clear and simplified genotyping between the wild-type and KO *Myo7a* alleles. Like *shaker1* mice, the *Myo7a^–/–^* mice exhibit spinning, hyperactivity, and head tilting behaviors that are identifiable around postnatal day 14 when mouse pups become active and begin walking (data not shown). It was also observed that *Myo7a^–/–^* eyes did not dilate as easily/quickly as heterozygous and wild-type controls (data not shown). Complete KO of MYO7A expression in the eye (*n* = 3 eyes per genotype) and inner ear (*n* = 2–4 per genotype) of *Myo7a^–/–^* mice was confirmed with Western blot (Figures 1B,C). Heterozygous *Myo7a^+/–^* mice expressed MYO7A at approximately 60% of wild-type (C57BL/6J) in the eye, and approximately 40% of wild-type in the cochlea. Due to the presence of the *lacZ* gene in the KO allele, we also probed retinal extracts with α-lacZ. We did not detect lacZ expression in either *Myo7a^–/–^* or *Myo7a^+/–^* mice (data not shown).

### MYO7A Deficiency Leads to Sensorineural Hearing Loss and Degeneration of Cochlear Hair Cells

**FIGURE 2:**
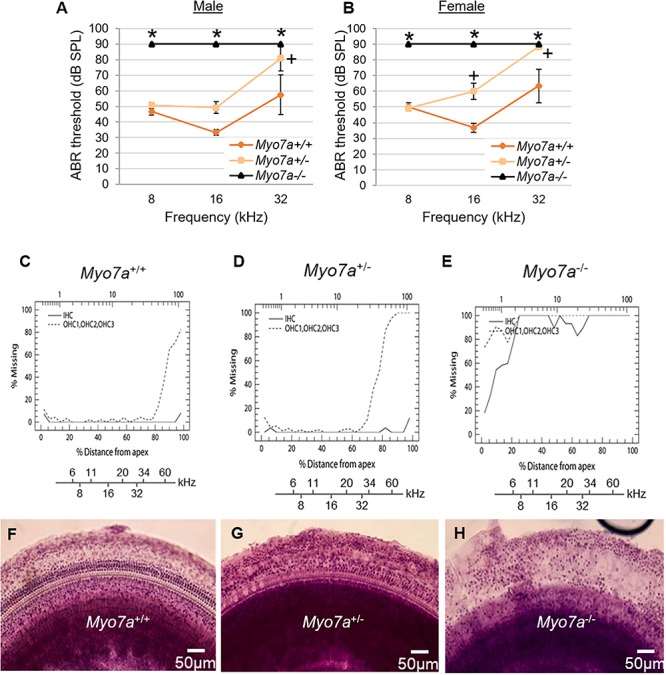
Characterization of cochlear hair cell structure and function in *Myo7a*^+/+^, *Myo7a*^+/–^, and *Myo7a*^–/–^ mice at 5 months of age. **(A,B)** Assessment of auditory brainstem response (ABR) thresholds in male **(A)** and female **(B)** mice were measured with a tone burst stimulus at 8, 16, and 32 kHz in (*n* = 5–6). Two-way ANOVA with Bonferroni’s multiple comparisons tests were used. Data are shown as means ± SEM. ^∗^*p* < 0.05 between *Myo7a*^–^*^/^*^–^ vs. *Myo7a*^+/+^; ^+^*p* < 0.05 between *Myo7a^+/–^* vs. *Myo7a^+/+^.* **(C–E)** Cochleograms were recorded in *Myo7a*^+/+^ **(C)**, *Myo7a^+/–^* **(D)**, and *Myo7a*^–^*^/^*^–^ **(E)** mice at 5 months of age. Graphs show percent loss of inner hair cells (IHC, solid line) and outer hair cells (OHC, dotted line) as a function of percent distance from the apex of the cochlea. Lower *x*-axes show the frequency-place map for mouse cochlea. **(F–H)** HC morphology in the middle turn of cochlear basilar membranes of *Myo7a*^+/+^ **(F)**, *Myo7a*^+/–^ **(G)**, and *Myo7a*^–/–^ **(H)** mice at 5 months of age.

To confirm that MYO7A deficiency affects hearing in this novel mouse model, ABR thresholds in *Myo7a*^+/+^, *Myo7a^+/–^*, and *Myo7a^–/–^* mice were measured at 5 months of age. Both *Myo7a^–/–^* males and females displayed profound hearing loss across all frequencies (Figures 2A,B). To our surprise, heterozygous *Myo7a^+/–^* mice also exhibited severe hearing loss at the mid and high frequencies. *Myo7a^+/–^* males displayed a 16–22-dB increase in ABR threshold at 16 and 32 kHz relative to age-matched *Myo7a*^+/+^ mice (Figure 2A), while *Myo7a^+/–^* females displayed a 23–25-dB increase in ABR threshold at 16–32 kHz compared to age-matched *Myo7a*^+/+^ mice (Figure 2B). Next, we investigated whether this loss of auditory function corresponded to a loss of cochlear HC structure. Cochleograms were prepared from *Myo7a*^+/+^, *Myo7a^+/–^*, and *Myo7a^–/–^* mice (Figures 2C–E). As expected, *Myo7a^–/–^* mice displayed near complete loss of both inner and outer HCs in the middle and basal cochlear regions (Figures 2E,H). While only minimal loss of IHC was observed in the apical and middle cochlear regions, *Myo7a^+/–^* mice displayed profound loss of OHC in the basal cochlea (Figures 2D,G) compared to wild-type littermates (Figures 2C,F).

**FIGURE 3:**
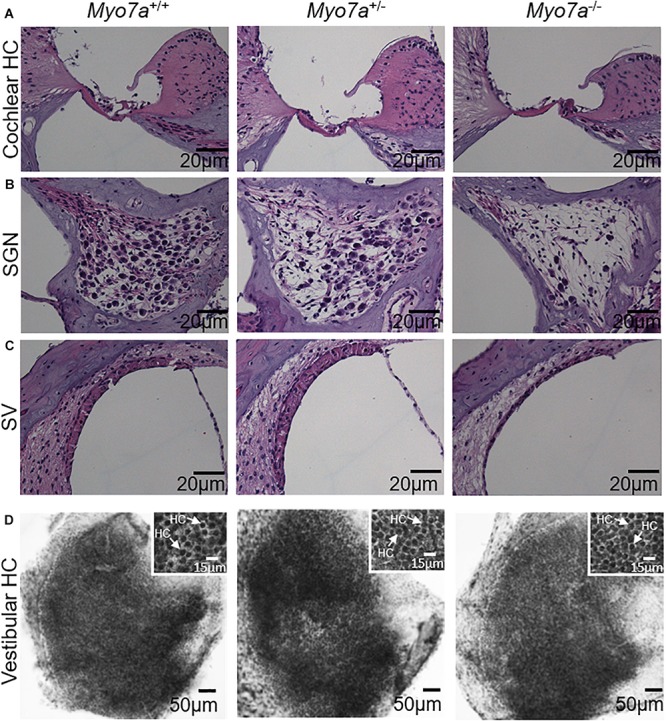
Assessment of basal–cochlear and utricle structure. Organ of Corti **(A)**, spiral ganglion neurons **(B)**, stria vascularis **(C)**, and vestibular hair cells **(D)** in 5-month-old *Myo7a*^+/+^, *Myo7a*^+/–^, and *Myo7a*^–/–^ mice. **(A–C)** Representative H&E-stained sections from the basal-cochlear region, imaged at 40× magnification. **(D)** Low magnification photomicrograph of representative hematoxylin-stained surface preparations of the macular utricle showing a dense array of vestibular hair cells covering the sensory epithelium (scale bar: 50 μm). Inset panels show a higher magnification view of densely packed vestibular hair cells with darkly stained nuclei (arrows, scale bar: 15 μm). SGN, spiral ganglion neuron; SV, stria vascularis. Scale bars for organ of Corti, SGN, and SV = 20 μm.

Cochlear pathology also manifests in SGNs and/or SV (Ding et al., 2016; White et al., 2017). To further investigate the impact of MYO7A deficiency on these tissues, histological examination was performed on H&E-stained cochlear sections. *Myo7a^–/–^* mice displayed near complete loss of HCs in the organ of Corti, SGNs, and reduced SV thickness or atrophy in the basal cochlea (Figures 3A–C). *Myo7a^+/–^* mice also displayed a reduction of SGNs in these regions, though not nearly to the extent seen in *Myo7a^–/–^* mice. Degeneration of cochlear structure in *Myo7a^–/–^* and *Myo7a^+/–^* mice was consistent with the reduced function observed in both genotypes. Taken together, we show that the absence or reduction of MYO7A results in the loss of cochlear HCs, SGNs, and SV, as well as a concomitant loss of hearing in young mice. This is the first report, to our knowledge, that a single functional *Myo7a* allele is insufficient to support normal hearing in mice. Instead, *Myo7a^+/–^* mice display characteristics of early onset age-related hearing loss which, in its early stages, typically manifests as a loss of threshold sensitivity at higher frequencies and a loss of outer HCs in the basal cochlea (Gates and Mills, 2005; Someya and Prolla, 2010).

### Vestibular HCs Are Preserved in *Myo7a^–/–^* Mice

Next, we investigated the impact of MYO7A on the structural integrity of vestibular HCs. Histological examination was performed on hematoxylin-stained HCs of the utricle macula of the vestibular tissue (Figure 3D). We did not observe any gross structural changes in the vestibular HCs of 5-month-old *Myo7a^–/–^* mice. Similarly, no obvious changes were observed in *Myo7a^+/–^* and *Myo7a*^+/+^ mice.

### MYO7A-Deficient Mice Do Not Recapitulate the Retinal Degeneration/Dysfunction Seen in USH1B Patients

**FIGURE 4:**
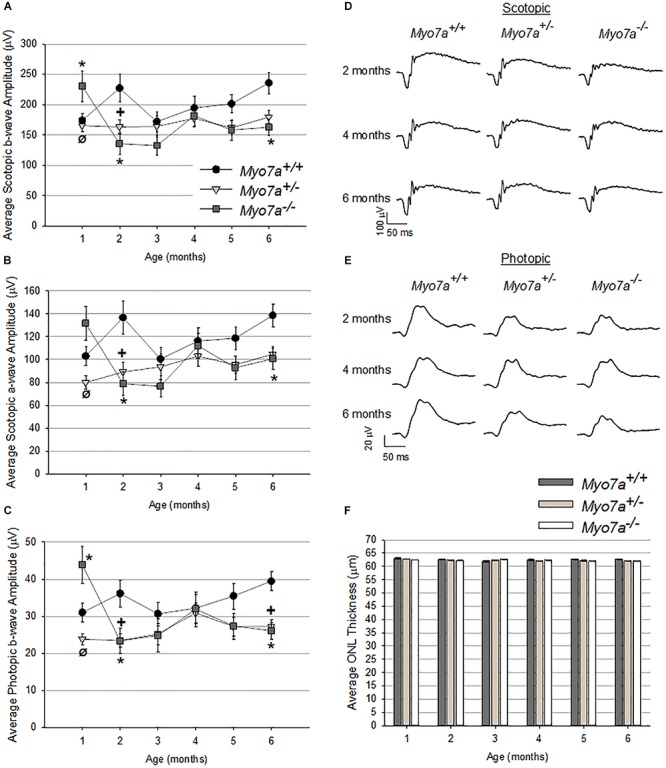
Characterization of retinal function and structure in *Myo7a*^+/+^, *Myo7a^+/–^*, and *Myo7a^–/–^* mice (*n* = 11–12 mice/genotype) over 6 months. Average scotopic b-wave **(A)**, scotopic a-wave **(B)**, and photopic b-wave **(C)** amplitudes. Equal numbers of males and females were used. Representative scotopic **(D)** and photopic **(E)** ERG waveforms at 2-, 4-, and 6-months of age. Scotopic and photopic values in panels **(A–D)** were recorded at 1.0 and 25.0 cd.s/m^2^, respectively. Average outer nuclear layer (ONL) thickness **(F)** in *Myo7a*^+/+^, *Myo7a^+/–^*, and *Myo7a^–/–^* mice over 6 months (*n* = 11–12 mice/genotype). Equal numbers of males and females were used. No gross changes in ONL thickness were observed. ^∗^*p* < 0.5 between *Myo7a^–/–^* vs. *Myo7a^+/+^*; ^+^*p* < 0.5 between *Myo7a^+/–^* vs. *Myo7a*^+/+^.

Retinal function and structure in age matched *Myo7a^–/–^*, *Myo7a^+/–^*, and *Myo7a*^+/+^ mice were assessed with ERG and OCT, respectively, every 30 days from 1 to 6 months of age. Scotopic and photopic amplitudes were only modestly reduced in both *Myo7a^+/–^* and *Myo7a^–/–^* mice relative to *Myo7a*^+/+^ controls after the 1 month time point (Figures 4A–E, photopic a-wave data not shown). By 6 months, the ERG amplitudes of *Myo7a^+/–^* and *Myo7a^–/–^* mice were reduced by approximately 20% that seen in *Myo7a*^+/+^ control mice. The marginal functional decreases observed in *Myo7a^+/–^* and *Myo7a^–/–^* mice were not associated with gross structural changes in the retina of these animals. OCT analysis confirmed that outer nuclear layer thickness (a measure of photoreceptor preservation) was maintained in all cohorts (Figure 4F).

Next, we evaluated the impact of MYO7A on RHO trafficking in *Myo7a^–/–^*, *Myo7a^+/–^*, and *Myo7a*^+/+^ mice. Previous studies employed immunogold labeling to demonstrate that MYO7A deficiency impaired/slowed transport of RHO through the photoreceptor connecting cilium (Liu et al., 1997, 1999). We used alternative methods to further investigate this phenomenon. *Myo7a^–/–^* mice were crossed with *RHOGFP*^+/+^ mice. The addition of GFP to the C-terminus of the RHO protein allows direct visualization of the protein and its expression is known to cause retinal degeneration (Chan et al., 2004). We hypothesized that, if RHO trafficking is impaired/slowed in the absence of MYO7A, then this phenotype may be compounded by the addition of a single *RHOGFP* allele and would be observable under fluorescent microscopy. We therefore generated *Myo7a^–/–^*:*RHOGFP^+/–^* mice by crossing *Myo7a^–/–^* and *RHOGFP*^+/+^ mice. To confirm that RHOGFP exacerbates degeneration in the presence of RHO mislocalization we also generated P23H^+^:*RHOGFP^+/–^* by crossing *RHOGFP*^+/+^ mice to transgenic *RHO*(P23H) mice as the P23H model exhibits mislocalized RHO as well as rapid degeneration (Illing et al., 2002; Mao et al., 2011). In all cases, mouse strains maintained one, wild-type *Rho* allele.

**FIGURE 5:**
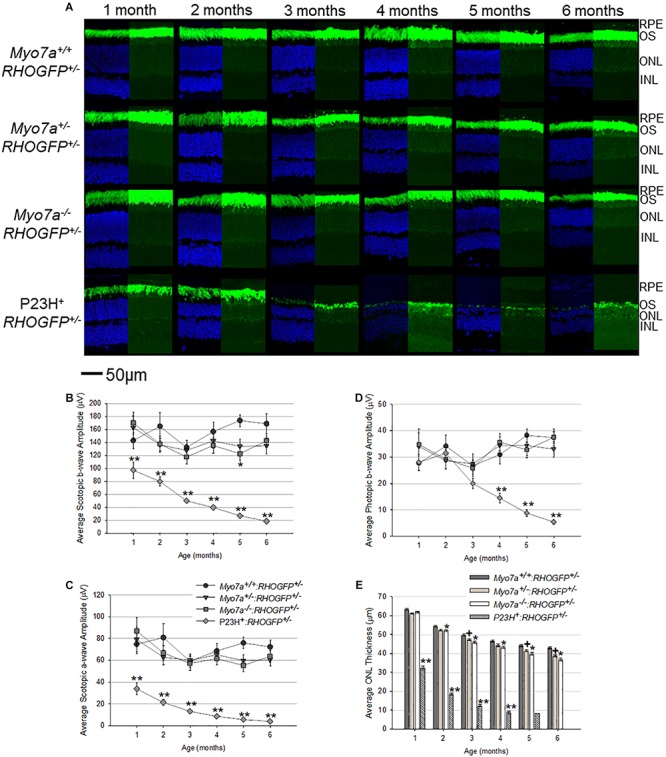
Characterization of retinal structure and function in *Myo7a^+/+^:RHOGFP^+/–^*, *Myo7a^+/–^:RHOGFP^+/–^*, *Myo7a^–/–^:RHOGFP^+/–^*, and P23H^+^:*RHOGFP^+/–^* mice (*n* = 11–12 mice/genotype) over 6 months. **(A)** Retinal cross sections (*n* = 4 mice/genotype) were co-stained with DAPI **(left)**. Endogenous GFP expression from RHOGFP in the same section (without DAPI) is shown **(right)**. Endogenous GFP expression on the right is overexposed for optimal visualization of RHOGFP localization in photoreceptor inner segments and ONL. All images taken at 60× magnification with identical exposure settings. Scale = 50 μm. Average **(B)** scotopic b-wave, **(C)** scotopic a-wave, and **(D)** photopic b-wave amplitudes in *Myo7a^+/+^:RHOGFP^+/–^*, *Myo7a^+/–^:RHOGFP^+/–^*, *Myo7a^–/–^:RHOGFP^+/–^*, and P23H^+^*:RHOGFP^+/–^* mice (*n* = 10–12 mice/genotype) over 6 months. Equal numbers of males and females were used. Scotopic and photopic averages were recorded at 1.0 and 25.0 cd.s/m^2^, respectively. **(E)** Average outer nuclear layer (ONL) thickness in *Myo7a^+/+^:RHOGFP^+/–^*, *Myo7a^+/–^:RHOGFP^+/–^*, *Myo7a*^–/–^*:RHOGFP^+/–^*, and P23H^+^*:RHOGFP^+/–^* mice (*n* = 10–12 mice/genotype) over 6 months. Equal numbers of males and females were used. No P23H^+^*:RHOGFP^+/–^* ONL data are included at 6 months because retinas were too thin for accurate measurement. ^∗^*p* < 0.5 between *Myo7a*^–/–^*:RHOGFP^+/–^* vs. *Myo7a^+/+^:RHOGFP^+/–^*; ^+^*p* < 0.5 between *Myo7a^+/–^:RHOGFP^+/–^* vs. *Myo7a^+/+^:RHOGFP^+/–^*; ^∗∗^*p* < 0.5 between P23H^+^:*RHOGFP^+/–^* vs. *Myo7a^+/+^:RHOGFP^+/–^*.

Rhodopsin–GFP fusion was imaged in retinal cross sections from all mouse strains (Figure 5A). RHOGFP mislocalization was not observed in *Myo7a^–/–^*:*RHOGFP^+/–^*, *Myo7a^+/–^*:*RHOGFP^+/–^*, and *Myo7a*^+/+^:*RHOGFP^+/–^* mice. In P23H^+^*:RHOGFP^+/–^* mice, however, mislocalization of RHOGFP in the photoreceptor inner segments was seen, especially at the 1- and 2-month time points before the onset of advanced degeneration.

Electroretinograms and OCT were performed on age-matched mice every 30 days from 1 to 6 months of age. Both *Myo7a^–/–^:RHOGFP^+/–^* and *Myo7a^+/–^:RHOGFP^+/–^* mice had slightly lower scotopic and photopic a- and b-wave amplitudes compared to *Myo7a^+/+^:RHOGFP^+/–^* controls, but this difference did not achieve statistical significance at most time points (Figures 5B–D). In contrast, the P23H^+^*:RHOGFP^+/–^* mice displayed significantly reduced ERG amplitudes at all time points and more rapid loss of retinal function relative to previous P23H + mouse models, a result presumably due to the addition of the *RHOGFP* allele (Price et al., 2011; Sakami et al., 2011; Mao et al., 2012). OCT analysis revealed slow, yet progressive, thinning in all *Myo7a:RHOGFP* mice (Figure 5E). This was expected as the fusion of GFP to the C-terminus of RHO was previously shown to cause degeneration (Chan et al., 2004). The ONLs of *Myo7a^+/–^:RHOGFP^+/–^* and *Myo7a^–/–^:RHOGFP^+/–^* mice were significantly thinner than *Myo7a^+/+^:RHOGFP^+/–^* controls by 2 months of age. MYO7A deficiency exacerbates the thinning caused by RHOGFP expression, as can be seen in the decreased ONL thickness in *Myo7a^–/–^:RHOGFP^+/–^* mice compared to *Myo7a^+/–^:RHOGFP^+/–^* mice (complete loss of MYO7A had a greater impact than partial loss). In contrast, photoreceptor degeneration in P23H^+^*:RHOGFP^+/–^* mice was rapid, with ONL thickness approximately 50% thinner relative to other strains at 1 month of age.

### MYO7A Is Differentially Expressed in Mouse vs. NHP

**FIGURE 6:**
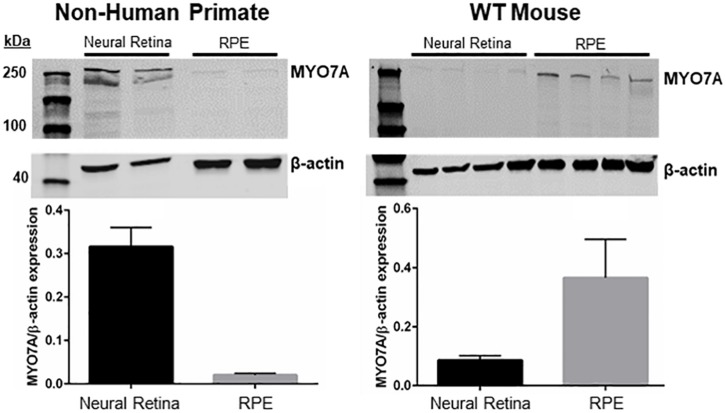
Expression of MYO7A in non-human primate vs. mouse. Neural retina and RPE from a 5-year-old male macaque and 5-month-old WT mice were manually separated. Protein from each compartment was immunoblotted with an antibody raised against MYO7A. The majority of MYO7A expression in macaque was found in neural retina **(left)**. The majority of MYO7A expression in mouse was found in RPE **(right)**. Protein levels were quantified and compared using *Fiji*. MYO7A expression was normalized to beta-actin. WT mouse = C57BL/6J.

MYO7A is expressed in the calyceal processes of primate photoreceptors, and these cells are the initial site of disease in USH1B patients (Jacobson et al., 2008, 2009, 2011; Sahly et al., 2012; Sumaroka et al., 2016; Subira et al., 2019). Mice potentially lack calyceal processes (Sahly et al., 2012) and do not exhibit retinal degeneration in the absence of MYO7A. One possible explanation for this discrepancy is that, unlike primates, mouse photoreceptors do not express MYO7A or rely on this protein to function. To investigate this further, we measured the relative expression of MYO7A in mouse vs. NHP photoreceptors and RPE via Western blot. The majority of MYO7A expression in adult (5-month-old) C57BL/6J mice is located in the RPE (Figure 6). In contrast, the majority of MYO7A expression is located in the neural retina of adult (5 years old) macaque (Figure 6). Because manual separation of the neural retina from the RPE is never 100% effective (some RPE cells remain attached to neural retina and vice versa), we cannot exclude the possibility that the minor signals observed in primate RPE or mouse neural retina were experimental artifact. This finding is consistent with previous reports that MYO7A is localized exclusively in mouse RPE, and not neural retina (el-Amraoui et al., 1996).

## Discussion

Our results demonstrate that *Myo7a^–/–^* mice, a newly established traditional KO model of USH1B, faithfully model the sensorineural hearing loss and degeneration of cochlear HCs, but not the robust retinal dysfunction/degeneration seen in patients. These findings are consistent with those previously described in other MYO7A-deficient mouse lines (Self et al., 1998; Libby and Steel, 2001; Lillo et al., 2003). A novel finding of this study is that *Myo7a^+/–^* mice also exhibited sensorineural hearing loss, degeneration of basal HCs in the cochlea, and marginal reductions in retinal function. This finding prompted us to look more deeply at previous reports of different *Myo7a* mutant mice for any evidence of haploinsufficiency.

Earlier studies that identified a role for MYO7A in cochlear development/function compared homozygous *Myo7a^*sh*1^*, *Myo7a^6*J*^*, and *Myo7a^816*SB*^* mice to heterozygous controls (no wild-type littermates were included) and thus haploinsufficiency was not interrogated (Self et al., 1998). The finding that MYO7A was necessary for migration of melanosomes into apical RPE was based on comparisons between *Myo7a^*sh*1–/–^* and *Myo7a^*sh*1+/–^* mice (Liu et al., 1998). Melanosome localization was reported to be normal in heterozygous mice, but no comparison to wild-type controls was performed (i.e., haploinsufficiency appears unlikely but was not fully interrogated). The conclusion that MYO7A was necessary for efficient RHO transport through photoreceptor connecting cilia was based on comparisons between homozygous and heterozygous *Myo7a^*sh*1^*, *Myo7a^4626*SB*^*, and *Myo7a^4494*SB*^* mice (Liu et al., 1999). Liu and colleagues concluded that opsin localization was normal in heterozygous mice, but no comparison to wild-type littermate controls was shown (i.e., haploinsufficiency appears unlikely but was not fully interrogated). The finding that MYO7A was required for normal phagocytosis of photoreceptor outer segments by RPE was based on comparisons between homozygous *Myo7a^4626*SB/*4626*SB*^* and heterozygous *Myo7a^+/4626*SB*^* mice (no wild-type littermate controls were used) (Gibbs et al., 2003). That study concluded that phagocytosis proceeded normally in heterozygous mice (i.e., haploinsufficiency appears unlikely but was not fully interrogated). Finally, the finding that MYO7A deficiency results in reduced retinal activity (∼20% reductions in ERG amplitudes) was based on comparisons between homozygous *Myo7a^4626*SB/*4626*SB*^* and heterozygous *Myo7a^+/4626*SB*^* mice (no wild-type littermate controls were used) and thus haploinsufficiency was not investigated (Libby and Steel, 2001; Colella et al., 2013). Taken together, previous studies did not explore whether heterozygous, carrier mice displayed an altered phenotype compared to “normal,” *Myo7a*^+/+^ mice.

To our knowledge, this is the first report of haploinsufficiency in mice carrying a single mutant *Myo7a* allele. This prompted us to search for evidence of cochlear/retinal dysfunction in USH1B carriers. Work from the 1970s showed that some Usher’s syndrome carriers exhibited (1) a tendency for hearing loss when compared to the normal population, (2) dark adaptation abnormalities, and (3) fundus abnormalities similar to retinitis pigmentosa (Dehaas et al., 1970; Holland et al., 1972; Sondheimer et al., 1979). These findings were associated with Usher’s syndrome as a whole, however, because genetic linkage had not yet been established. By the 1990s, USH1B had been linked to chromosome region 11p13-15 (Kimberling et al., 1992; Smith et al., 1992; Bonnetamir et al., 1994; Weil et al., 1995) and studies found that a number of obligate carriers exhibited significant audiological and ophthalmological abnormalities (Wagenaar et al., 1995). These clinical findings are intriguing, and align with our findings in heterozygous *Myo7a^+/–^* mice, but a more comprehensive assessment of genetically confirmed USH1B carriers is warranted to determine their significance.

Age-related hearing loss, or presbycusis, typically begins with a loss of sensitivity to high-frequency sounds and a loss of outer HCs in the basal cochlea (Gates and Mills, 2005). Over time, these deficits progress toward lower-frequencies and more apical regions of the cochlea. The loss of high-frequency ABR thresholds and basal cochlear HCs we observed in 5-month-old *Myo7a^+/–^* mice appear to mimic the early stages of presbycusis. Longer-term analysis is needed to determine whether sensorineural hearing loss and degeneration of cochlear structure in heterozygous mice worsens over time. Recently, it was reported that high frequency hearing loss occurred in rats with reduced estrogen levels (as a result of ovariectomy) and that this loss was associated with decreases in MYO7A expression (Diao et al., 2019). Investigators hypothesize that reduced levels of estrogen and MYO7A are responsible for the hearing loss observed in post-menopausal women (Svedbrant et al., 2015; Zhang et al., 2018). Notably, the hearing loss we observed in heterozygous *Myo7a^+/–^* mice was more pronounced in females at high frequencies.

While MYO7A localization in cochlear tissues and its role in auditory function are well-established (Hasson et al., 1995; Hosoya et al., 2016), its impact on the vestibular system has been less studied. In the vestibular system, MYO7A is expressed in the stereocilia, cuticular plates, and cell bodies in macular and semicircular canal HCs of mice and guinea pigs (Hasson et al., 1997) and clearly has a functional role in the vestibular system because MYO7A-deficient mice manifest behaviors (circling, head tilting) associated with its dysfunction. Our results show that, unlike cochlear HCs which are almost completely degenerated, vestibular HCs are present in *Myo7a^–/–^* mice at 5 months of age. Similar results were seen in *Myo7a^*I*487*N/I*487*N*^ ewaso* mice, the only other MYO7A-deficient mouse line in which vestibular HC structure has been described (Miller et al., 2012). Rows of stereocilia are missing from the vestibular HCs and disrupted staircase morphology were observed in this mouse (Miller et al., 2012). However, the vestibular HCs were still present and contained stereocilia of various lengths. These findings shed light on results obtained in gene replacement studies recently conducted in other USH mouse models wherein vestibular HCs proved more amenable to therapy than cochlear HCs (Emptoz et al., 2017; Isgrig et al., 2017). Although our results do not rule out ultrastructural defects that may exist in MYO7A-deficient vestibular HCs, they suggest that these cells either do not degenerate, or are lost at a slower rate than their cochlear counterparts in the absence of MYO7A and support further analysis of vestibular HC structure in USH1B patients. If vestibular HCs are maintained, there exists the potential to treat USH1B-associated vestibular dysfunction via gene supplementation. This is an attractive prospect as currently available treatments for hearing loss (cochlear implants) and planned treatments for vision loss (gene supplementation) will not alleviate the loss of equilibrium caused by dysfunction of the vestibular system.

USH1B patients can retain both central and peripheral islands of structurally and functionally intact photoreceptors, suggesting that they may be good candidates for retinal gene therapy (Jacobson et al., 2008, 2009, 2011; Sumaroka et al., 2016). Toward that end, various studies, including ours, have been conducted to determine whether MYO7A-deficient mice have ocular phenotypes that are sufficiently robust to serve as outcome measures in preclinical gene therapy studies. Like previously examined mouse models, we show that *Myo7a^–/–^* do not exhibit retinal degeneration and have only marginal decrements in retinal function. Contrary to previous reports that MYO7A deficiency in homozygous *Myo7a^*sh*1^*, *Myo7a^4494*SB*^*, and *Myo7a^4626*B*^* mice led to impaired/slowed transport of RHO through the photoreceptor connecting cilium, we did not observe accumulation of RHOGFP in the inner segments of photoreceptors in *Myo7a^–/–^:RHOGFP^+/–^* mice (Liu et al., 1999). OCT scans of USH1B patients suggest that the physiopathologic basis of this disease occurs at the level of the photoreceptors (Jacobson et al., 2008, 2011; Sumaroka et al., 2016; Subira et al., 2019). Specific findings include disruption/loss of the external limiting membrane, disruption of the myoid zone and ellipsoid zone, and loss of the outer segments. None of these disruptions were observed in OCT scans of *Myo7a*^–/–^ or *Myo7a*^+/–^ mice (data not shown). Our results, and others (Sahly et al., 2012), suggest this discrepancy is due to differential expression of MYO7A/photoreceptor structure across species. The majority of primate MYO7A is expressed in neural retina (not RPE), whereas it is marginally detected in mouse neural retina but expresses strongly in mouse RPE. These results argue that MYO7A-deficient mice are of limited value for elucidating the mechanisms underlying the retinal degeneration/dysfunction seen in USH1B patients. However, they do faithfully mimic the auditory and vestibular phenotypes and thus could reasonably be used to study these aspects of the disease and potential therapeutic interventions targeted to the inner ear. In addition, the observation that MYO7A haploinsufficiency impacts auditory function in mice warrants further investigation in USH1B carriers.

Due to the large size of the *MYO7A* cDNA, dual AAV vector-based strategies are currently being developed to address USH1B (Colella et al., 2013; Lopes et al., 2013; Dyka et al., 2014). While the novel *Myo7a^–/–^* mice described herein are unlikely be useful for measuring therapeutic efficacy of these therapies in retina (no sufficient outcome measures are available), they will be instrumental in evaluating the efficiency of dual vector platforms because they are definitively null, and can be easily genotyped/maintained. The best performing platforms will be defined in part by their propensity to drive full-length MYO7A expression in *Myo7a^–/–^* retina. Ultimately, these dual vectors will be best evaluated for their ability to recapitulate endogenous MYO7A expression in primate retina.

## Data Availability Statement

The datasets generated for this study are available on request to the corresponding author.

## Ethics Statement

The animal study was reviewed and approved by the University of Florida’s Institutional Animal Care and Use Committee.

## Author Contributions

KC performed the experiments, analyzed the data, and wrote the initial draft of the manuscript. DF, JP, WL, and SC performed the experiments. SMC analyzed the data. M-JK, DD, RS, and SS designed and performed the experiments, and analyzed the data. SEB and SLB designed and supervised the project and edited the manuscript.

## Conflict of Interest

The authors declare that the research was conducted in the absence of any commercial or financial relationships that could be construed as a potential conflict of interest.
